# Link Between m6A Modification and Cancers

**DOI:** 10.3389/fbioe.2018.00089

**Published:** 2018-07-13

**Authors:** Zhen-Xian Liu, Li-Man Li, Hui-Lung Sun, Song-Mei Liu

**Affiliations:** ^1^Center for Gene Diagnosis, Zhongnan Hospital of Wuhan University, Wuhan, China; ^2^Department of Chemistry and Institute for Biophysical Dynamics, Howard Hughes Medical Institute, University of Chicago, Chicago, IL, United States

**Keywords:** m6A, mRNA, cancers, function, structures

## Abstract

N6-methyladenosine (m6A) epitranscriptional modification has recently gained much attention. Through the development of m6A sequencing, the molecular mechanism and importance of m6A have been revealed. m6A is the most abundant internal modification in higher eukaryotic mRNAs, which plays crucial roles in mRNA metabolism and multiple biological processes. In this review, we introduce the characteristics of m6A regulators, including “writers” that create m6A mark, “erasers” that show demethylation activity and “readers” that decode m6A modification to govern the fate of modified transcripts. Moreover, we highlight the roles of m6A modification in several common cancers, including solid and non-solid tumors. The regulators of m6A exert enormous functions in cancer development, such as proliferation, migration and invasion. Especially, with the underlying mechanisms being uncovered, m6A and its regulators are expected to be the targets for the diagnosis and treatment of cancers.

## Introduction

More than 100 kinds of chemical modifications of RNA have been identified in living organisms (Boccaletto et al., 2018). Studies have widely reported certain types of RNA modifications in eukaryotic mRNA, including N1-methyladenosine (m1A), N6-methyladenosine (m6A) and 5-methylcytosine (m5C), among which m6A was first discovered in the 1970s. m6A is the most abundant internal modification of mRNA and long noncoding RNA (lncRNA) in the majority of eukaryotes. Besides, m6A significantly clusters around the stop codon and 3′ untranslated region (3′UTR) (Dominissini et al., 2012; Meyer et al., 2012; Bodi et al., 2015). m6A modification mostly occurs at RRACH motif (R denotes A or G, H denotes A, C, or U) (Narayan and Rottman, 1988; Csepany et al., 1990; Narayan et al., 1994).

**Figure 1:**
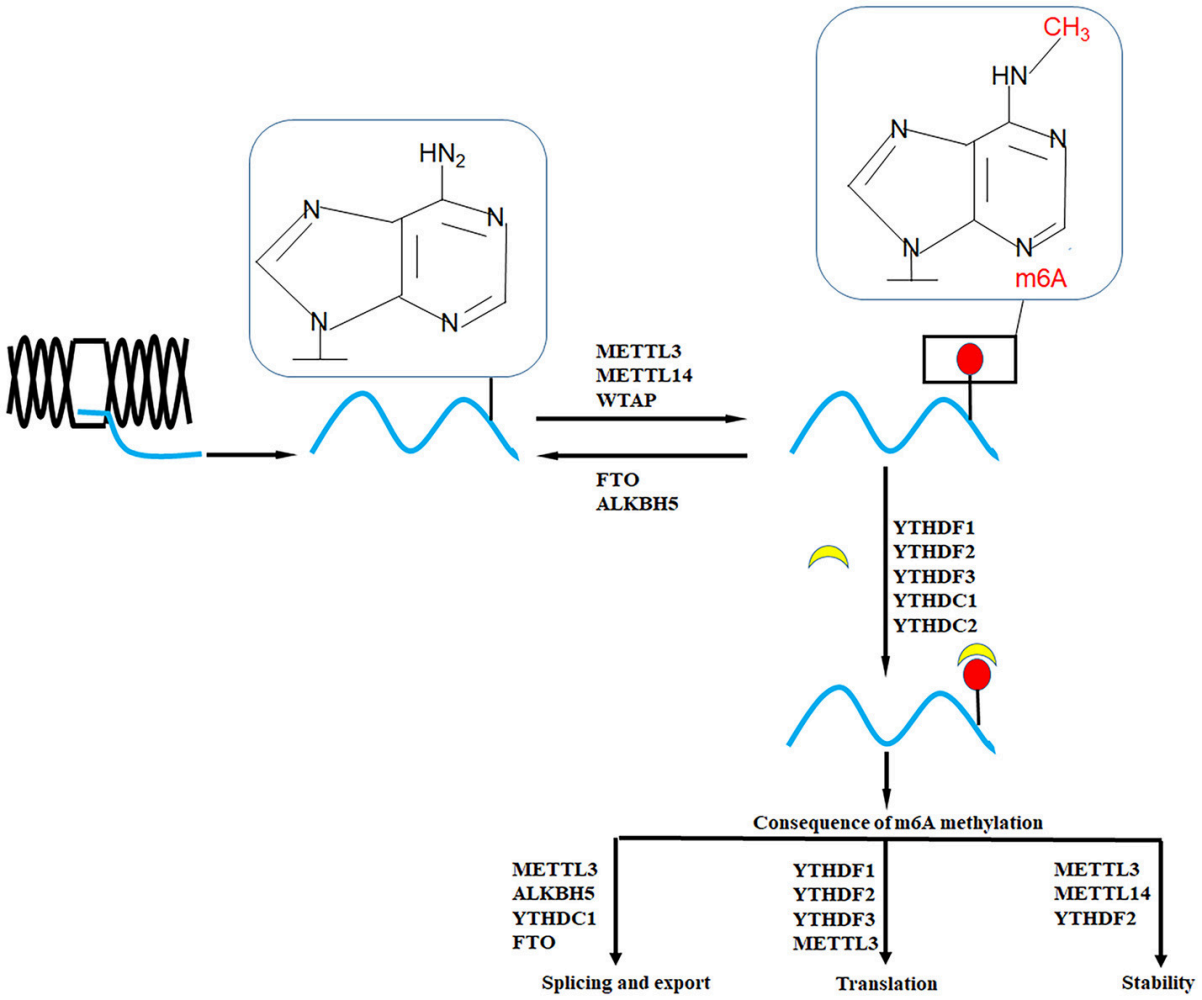
The establishment and function of m6A RNA methylation. The m6A of RNA methylation is dynamically regulated by “writers” (METTL3, METTL14, WTAP, and others) and “erasers” (FTO and ALKBH5). “readers” (YTHDF1, YTHDF2, YTHFDF3, YTHDC1, and YTHDC2) are binding proteins of m6A. Different regulators of m6A have different functions on transcriptional process, including splicing, export, translation, and stability.

As shown in Figure 1, the formation of m6A is a reversible process (Jia et al., 2013), m6A “writers” with methyltransferase activity are consisted of three individual proteins: methyltransferase-like 3 (METTL3), methyltransferase-like 14 (METTL14), and Wilms' tumor 1-associating protein (WTAP). Obesity-associated protein (FTO) and alkB homolog 5 (ALKBH5) are m6A demethylase (Jia et al., 2011; Zheng et al., 2013). Another protein family is m6A “readers,” which can recognize m6A modification to modulate mRNA fate (Li et al., 2017a).

m6A modification regulates mRNA at different levels, including structure, maturation, stability, splicing, export, translation and decay (Liu and Zhang, 2018). Moreover, m6A is also involved in cell fate decision, cell cycle regulation, cell differentiation and circadian rhythm maintenance (Wu et al., 2018). Furthermore, multiple RNA binding proteins that are affected by m6A. For example, heterogeneous nuclear ribonucleoprotein G (HNRNPG), a new m6A reader protein, utilizes a low-complexity region to recognize a motif exposed by m6A modification (Liu et al., 2017). HNRNPC and HNRNPA2B1 are two abundant nuclear RNA-binding proteins (Dai et al., 2018). Currently, increasing evidence has shown the roles of m6A in human diseases. Here, we summarized the functions and roles of m6A regulators in diverse cancers (Wang et al., 2017a), such as acute myeloid leukemia (AML), glioblastoma (GBM), lung cancer, liver cancer, hoping to elucidate the contributions of m6A in cancer process.

## Proteins involved in the M6A methylation

### m6A “writers”

METTL3, METTL14, and WTAP form m6A methyltransferase complex (Bokar et al., 1997; Liu et al., 2014; Ping et al., 2014). METTL3, a 70-kDa key protein, is firstly identified as m6A “writer” (Bokar et al., 1997). A recent study has indicated that knockdown of METTL3, METTL14, and WTAP could decrease m6A level in polyadenylated RNA. The gel filtration experiment has revealed that METTL3 and METTL14 form a stable METTL3-14 complex with a 1:1 stoichiometric ratio, then WTAP binds to the METTL3-14 complex (Liu et al., 2014). Further crystallization and structure determination have demonstrated that METTL3-14 heterodimer is asymmetric. In this crystallized METTL3-14 complex, both METTL3 and METTL14 contain MTA-70 methyltransferase domain, two CCCH-type zinc finger motifs exist in the N-terminal region of METTL3 and an N-terminal extension presents in the N-terminal region of METTL14 (Sledz and Jinek, 2016). Additionally, METTL3 acts as the catalytic core, transferring methyl group from S-adenosylmethionine (SAM) to acceptor adenine moiety. METTL14 serves as the RNA-binding platform, promoting the binding of RNA substrate and enhancing the complex integrity (Wang et al., 2016a,b). METTL3-14 complex dimer induces m6A deposition on nuclear RNA. WTAP does not possess methyltransferase activity (Ping et al., 2014), however, it interacts with METTL3-14 complex to affect m6A methyltransferase activity *in vivo* and localization in nuclear speckles (Liu et al., 2014).

### m6A “erasers”

FTO and ALKBH5 have been reported to exhibit demethylation activity (Jia et al., 2011; Zheng et al., 2013). The level of m6A in mRNA increases after FTO knockdown, while m6A in mRNA notably decreases after overexpression of the wild-type FTO (Jia et al., 2011). However, Meyer C et al. reported that, compared with m6A, FTO showed higher affinity with the modification N6, 2'-O-dimethyladenosine (m6A_m_), a reversible modification influencing cellular mRNA fate. FTO preferentially demethylated m6A_m_ and reduced the stability of m6A_m_ mRNA (Mauer et al., 2017). The difference across the studies may be caused by the location of FTO in different cell lines. ALKBH5 localizes in the nucleus, the level of m6A in mRNA significantly decreases in ALKBH5 overexpressed cells (Zheng et al., 2013). ALKBH5 strictly selects substrate in catalysis progression, and the loss of ALKBH5 impairs RNA metabolism, mRNA export and assembly (Zheng et al., 2013).

### m6A “readers”

Remarkably, m6A regulates gene expression through m6A “readers,” a group of various proteins which can recognize m6A modification. The highly conserved YT521-B homology YTH domain family proteins include YTHDF1, YTHDF2, and YTHDF3 in the cytoplasm, and YTH domain containing 1 (YTHDC1) in the nucleus (Wang et al., 2014a, 2015; Xu et al., 2014; Xiao et al., 2016; Shi et al., 2017). YTHDF1 promotes the translation of m6A-methylated mRNA, YTHDF2 accelerates the decay of m6A-methylated mRNA, and YTHDF3, together with YTHDF1 and YTHDF2, noticeably enhances the metabolism of m6A-methylated mRNA in the cytoplasm (Shi et al., 2017). In nuclear speckle localization, YTHDC1 influences mRNA splicing by facilitating SRSF3 but inhibiting SRSF10 (Roundtree et al., 2017). Although YTHDC1 knockdown does not significantly alter the distribution of non-target transcripts, it can affect mature mRNA transport from the nucleus to the cytoplasm (Xiao et al., 2016). YTH domain containing 2 (YTHDC2) can preferentially bind to m6A-containing transcripts resulting in the decrease of mRNA abundance and the enhancement of translation efficiency via the interaction with translation initiation and decay machineries (Hsu et al., 2017).

## Consequence of m6A methylation

### Impact of m6A on splicing and export

mRNA is spliced into mature transcript and exported from nucleus to the cytoplasm, then they can be translated into proteins. Therefore, mRNA nuclear export is a vital step which connects transcription with translation. Simultaneously, mRNA export also regulates gene expression (Wickramasinghe and Laskey, 2015). Depletion of METTL3 delays the export of mature mRNA. In addition, circadian period shows elongation by prolonging nuclear retention of mature mRNA of the clock genes Per2 and Arntl (Fustin et al., 2013). ALKBH5 plays vital roles in mRNA export as well as roles in RNA metabolism and the association of the nuclear speckle proteins (Zheng et al., 2013). YTHDC1 can interact with SRSF3, a nuclear export adaptor protein and splicing factor, to modulate the binding of RNA with SRSF3 and NXF1, then SRSF3-NXF1 and YTHDC1-SRSF3 protein complexes lead m6A-modified mRNA into export pathway (Roundtree et al., 2017). FTO mediates nuclear pre-mRNA alternative splicing. For instance, FTO controls alternative splicing of RUNX1T1, an adipogenesis-related transcription factor, affecting adipogenesis (Zhao et al., 2014). Furthermore, FTO preferentially binds to pre-mRNA in intronic regions, and FTO knockdown leads the substantial changes in pre-mRNA splicing with exon skipping events (Bartosovic et al., 2017).

### Impact of m6A on translation

Translation is regulated by m6A modification through several mechanisms. YTHDF1 is known to promote translation efficiency by binding with m6A. The translation efficiency decreases notably after YTHDF1 knockdown. YTHDF1 ensures the efficient protein production from m6A modification transcript (Wang et al., 2015). In addition, YTHDF1 recruits eukaryotic initiation factor 3 (eIF3) to directly bind a single m6A modification in the 5′UTR, which facilitates ribosome loading and recruits the 43S complex to promote translation (Meyer et al., 2015).

According to previous studies, m6A sites are mainly enriched in the stop codon and 3'UTR (Dominissini et al., 2012; Meyer et al., 2012). Interestingly, heat shock stress induces an elevated m6A peak in the 5'UTR. Upon heat shock stress, the nuclear YTHDF2 limits the demethylation of m6A “eraser” FTO to preserve 5'UTR methylation of stress-induced transcripts. The increased m6A-marked 5'UTR methylation promotes cap-independent translation initiation, providing a mechanism for selective mRNA translation under heat shock stress (Zhou et al., 2015). In translational process, YTHDF3 interacts with ribosomal 40S/60S subunits and significantly enhances translation efficiency of YTHDF1 and YTHDF3 sharing targeting m6A-methylated mRNA (Li et al., 2017a).

METTL3 also directly promotes translation of certain mRNA in human cancer cells by recruiting eIF3. Reader proteins YTHDF1, YTHDF2 and binding-partners METTL14, WTAP are independent of translational control of METTL3 (Lin et al., 2016).

### Impact of m6A on RNA stability

The stability of RNA is closely associated with m6A-dependent degradation process. Knockout of METTL3 and METTL14 in embryonic stem cells can reduce mRNA decay, leading to increase of target mRNA expression, resistance of differentiation, enhancement of self-renewal and maintenance of the pluripotency state of embryonic stem cell (ESC) (Liu et al., 2014; Wang et al., 2014b). A RNA-binding protein, human antigen R (HuR), can increase RNA stability, however, m6A modification inhibits the binding ability by interfering with the HuR (Srikantan et al., 2012).

YTHDF2 directly modulates mRNA decay pathway in an m6A-dependent way (Wang et al., 2014a). Compared to the unmethylated mRNA, YTHDF2 has about 16-fold higher binding affinity to m6A-marked mRNA, showing significant increase of decay rate and shorter half-life (Batista et al., 2014; Fu et al., 2014). The carboxy-terminal domain of YTHDF2 selectively binds to methylated mRNA and leads to YTHDF2-bound transcripts to decay sites (Wang et al., 2014a). Furthermore, YTHDF2 N-terminal region interacts with the SH domain of the CNOT1 subunit recruiting the CCR4-NOT complex to accelerate the deadenylation of YTHDF2-bound m6A-containing mRNA (Du et al., 2016).

### Roles of m6A methylation in cancers

**Figure 2:**
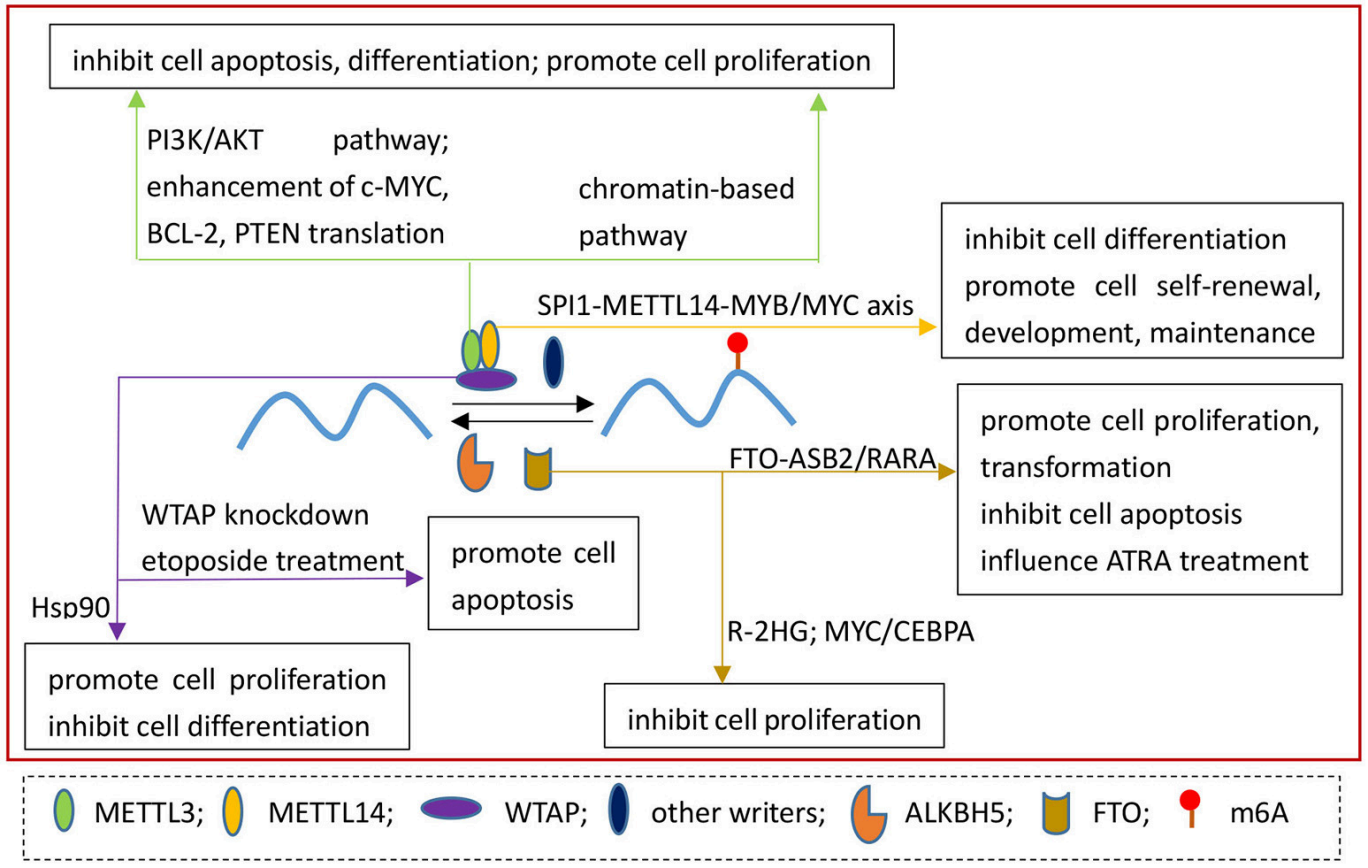
The roles of m6A regulatory proteins in AML.

**Figure 3:**
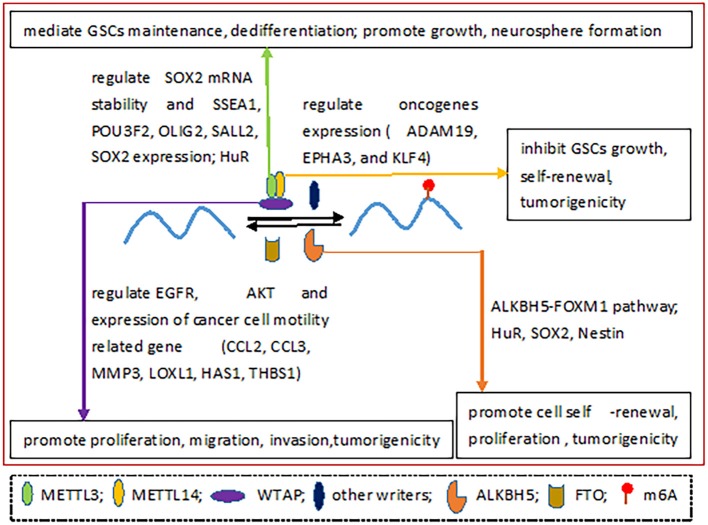
The roles of m6A regulatory proteins in GBM.

**Figure 4:**
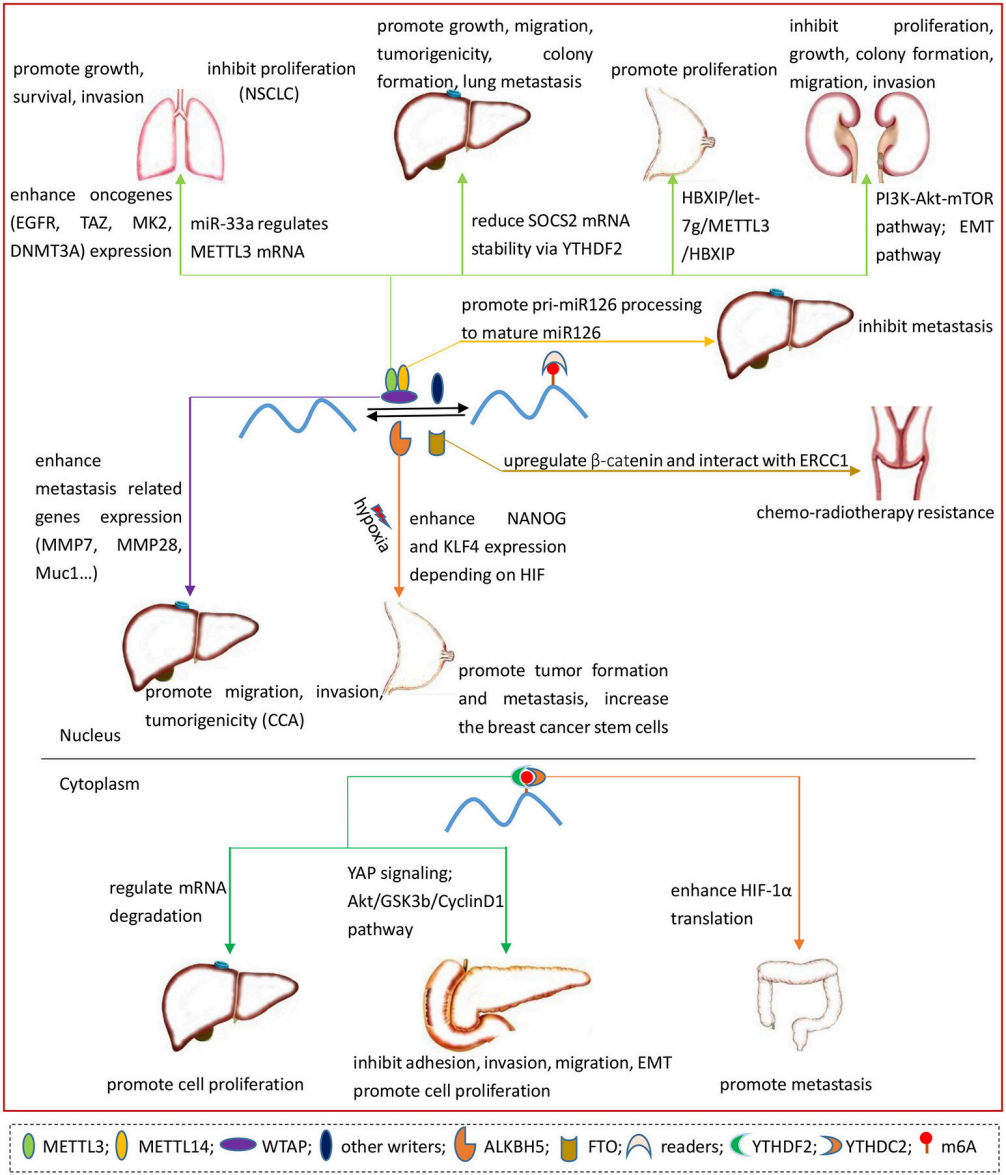
The roles of m6A regulatory proteins in different cancers, including lung cancer, NSCLC, HCC, CCA, breast cancer, RCC, pancreatic cancer, colon cancer, cervical cancer.

Currently, increasing studies focus on the potential links between m6A and cancers. In different tumors, the effect of m6A modification could be different. The changes of m6A also affect the progression of tumors, including proliferation, growth, invasion and metastasis (Figures 2–**4**).

### Acute myeloid leukemia (AML)

The common types of leukemia are acute lymphoblastic leukemia (ALL), chronic lymphocytic leukemia (CLL), acute myeloid leukemia (AML) and chronic myeloid leukemia (CML). As regard the incidence and mortality, AML ranks the first in hematopoietic malignancies. Moreover, different cytogenetic abnormalities [t(8;21)(q22;q22), inv(16)(p13q22)/t(16;16)(p13;q22), t(15;17)(q22;q11~21), and abn(11q23)] and molecular abnormalities (FLT3-ITD, MLL PTD and NPM1 mutations) show different pathogenesis (Frohling et al., 2005). Although new therapies for AML, such as epigenetic targeted drugs and immunotherapies (Yang and Wang, 2018), have been developed, the survival rates have not improved significantly.

In recent years, the molecular mechanism of m6A “writers” and “erasers” in leukemia has been explored (Figure 2). METTL3 expression was increased in AML patients and plays an oncogenic role. METTL3 inhibits cell differentiation and apoptosis, and promotes cell proliferation through increasing c-MYC, BCL-2, PTEN translation. MELLT3 also activate PI3K/AKT pathway to control cell differentiation and self-renewal (Vu et al., 2017). Interestingly, METTL3 works on a chromatin-based pathway independently of METTL14 by localizing to the transcriptional start sites of active genes through CAATT-box binding protein (CEBPZ), which resulting in an increase of translation of the corresponding mRNA (Barbieri et al., 2017). In addition, almost all members of “writers” are overexpressed in AML, including METTL3, METTL14, WTAP, and KIAA1429. METTL14 plays an inhibitory role in normal myelopoiesis, whereas suppresses cell differentiation in AML. In SPI1-METTL14-MYB/MYC axis, METTL14, is downregulated by SPI1, exerts an oncogenic role in enhancing leukemia stem/initiating cells self-renewal and repressing myeloid differentiation by regulating MYB and MYC via m6A modification (Weng et al., 2017). WTAP, another member of m6A writers, has been shown an association with AML. Elevated WTAP promotes cell proliferation and inhibits cell differentiation of AML. WTAP also directly links with Hsp90 (Bansal et al., 2014), a molecular chaperone maintains the stability of many tumor-promoting oncoproteins (Whitesell and Lindquist, 2005). More importantly, the knockdown of WTAP with etoposide treatment significantly promotes apoptosis. However, without etoposide treatment, this phenomenon doesn't occur (Bansal et al., 2014). These outcomes suggest WTAP is a potential target for the treatment of AML.

Although both FTO and ALKBH5 have no effect on AML patients' survival rates (Vu et al., 2017), in certain subtypes of AML, such as MLL-rearranged AML, acute promyelocytic leukemia (APL), t(11q23) and t(15;17) AMLs, FTO is highly expressed. FTO reduces the m6A levels of ASB2 and RARA by targeting their UTRs and affects all-trans-retinoic acid (ATRA) treatment efficiency (Li et al., 2017c). R-2-hydroxyglutarate(R-2HG), which accumulates in isocitrate dehydrogenase 1/2 (IDH1/2) mutant cancers, can increase global m6A via inhibiting FTO, resulting in the decrease of MYC/CEBPA mRNA stability and inhibition of leukemia proliferation (Su et al., 2018).

Genetic alterations of m6A regulatory genes including “writers,” “readers,” and “erasers” have indicated an inferior cytogenetic risk in AML by analyzing datasets from the Cancer Genome Atlas Research Network (TCGA) (Kwok et al., 2017). METTL3, METTL14, WTAP FTO and R-2HG, interacting with FTO, are potential therapeutic targets for AML. However, study on the “readers” of m6A has not yet been reported.

### Glioblastoma (GBM)

Glioblastoma [GBM; WHO grade IV (Xi et al., 2016)] is an invasive malignant primary brain tumor with a median survival of 10–11 months and a poor quality of life(Rick et al., 2018). With the development of epigenetics, the correlation of m6A with GBM has been uncovered in recent years (Figure 3).

In GBM, the level of m6A on mRNA is reduced, however, when glioblastoma stem-like cells (GSCs) are induced into differentiation, the m6A level is increased. The effect of knockdown METTL3 or METTL14 on promoting GSCs growth and self-renewal have been further confirmed by overexpression experiments. Additionally, depletion of METTL3 or METTL14 enhances tumor tumorigenicity while FTO inhibitor treatment prevents tumor deterioration. What's more, knockdown of METTL3 or METTL14 alters gene expression leading to elevation of oncogenes, including ADAM19, EPHA3, and KLF4. FTO inhibitor MA2 can prolong the life span of GSCs transplanted animals, suggesting m6A is expected to be a target for the treatment of GBM (Cui et al., 2017). m6A modification related genes and pathways could serve as promising molecular targets for GBM treatment.

A study has proved that METTL3 increases, METTL14 and ALKBH5 decrease, whereas FTO has no significant change in GSCs. METTL3 mediates GSCs maintenance and dedifferentiation by regulating the stability of the SOX2 mRNA though installing m^6^A on the SOX2-3′UTR. The complete structures of METTL3 and HuR are vital to this procedure. Furthermore, suppressed METTL3 can result in the inhibition of GSC growth and the neurosphere formation, the reduction of stem cell-specific marker (SSEA1) and glioma reprogramming factors (including POU3F2, OLIG2, SALL2, and SOX2) expression. In particular, SOX2 mRNA has a high affinity to METTL3. Crucially, depletion of METTL3 leads to an increase of radiation sensitivity and a decrease of DNA repair, providing a direction for overcoming radiation tolerance (Visvanathan et al., 2018).

WTAP is overexpressed in GBM. WTAP enhances cell proliferation, migration, invasion and tumorigenicity of glioblastoma cells in xenograft via mediating phosphorylation of epidermal growth factor receptor (EGFR) and AKT. Besides, WTAP regulates the expression of certain genes related to motility of cancer cells, such as chemokine ligand 2 (CCL2), chemokine ligand 3 (CCL3), matrix metallopeptidase 3 (MMP3), lysyl oxidase-like 1 (LOXL1), hyaluronan synthase 1 (HAS1), and thrombospondin 1 (THBS1) (Jin et al., 2012). The high expression of WTAP is an independent negative prognostic factor that is associated with age and WHO grade, predicting poor overall survival for GBM patients (Xi et al., 2016). Therefore, WTAP may be a prognostic marker for GBM.

Contrary to the text mentioned above (Visvanathan et al., 2018), ALKBH5 is elevated in GSCs (Zhang et al., 2017), enhancing cell self-renewal, proliferation and tumorigenicity. ALKBH5 serves as a poor prognostic indicator in patients with glioma. ALKBH5 removes m^6^A from FOXM1 (which is a transcription factor and is highly expressed in GBM patients) nascent transcripts by binding to the 3′UTR, enhancing FOXM1 expression. This process can be strengthened by a long non-coding RNA antisense to FOXM1 (FOXM1-AS). Depletion of ALKBH5 and FOXM1-AS inhibits GSCs tumorigenesis via the FOXM1 axis. In addition, certain evidence has confirmed that HuR, SOX2 and Nestin also play a crucial role in this process (Zhang et al., 2017).

### Lung cancer

Lung cancer includes small cell lung carcinoma (SCLC) and non-small-cell lung carcinoma (NSCLC), and NSCLC accounts for approximately 85% of all cases. Although the incidence and death rate have declined, the 5-year survival rates remain poor (Molina et al., 2008).

As seen in Figure 4, METLL3 enhances the translation of certain oncogenes such as EGFR, TAZ, MAPKAPK2 (MK2), and DNMT3A by recruiting eIF3 to the translation initiation complex which is independent of METTL3 catalytic activity and m6A readers. Furthermore, METTL3 plays a driving role in cancer cell growth, survival and invasion (Lin et al., 2016). Another study shows that miR-33a inhibits the proliferation of NSCLC cells by binding to the 3′UTR of METTL3 mRNA (Du et al., 2017). The links between microRNA, METTL3, and NSCLC suggest METTL3 may be a novel target for NSCLC therapy.

### Liver cancer: hepatocellular carcinoma (HCC) and cholangiocarcinoma (CCA)

#### Hepatocellular carcinoma (HCC)

The majority of liver cancer is HCC. The incidence and mortality of HCC increase per year owing to the lack of precision diagnosis at an early-stage, and prediction of tumor metastasis and postsurgical recurrence. The 5-year survival rate is only 18%, thus the mechanism and pathogenesis of HCC are urgent to be addressed (Ma et al., 2017). Increasing evidence has shown that m6A and regulators are critical for the development of liver cancer (Figure 4).

Currently, only two researches have revealed the association of m6A “writers” with HCC, focusing on METTL3 and METTL14, respectively. METTL3, increasing in HCC, can facilitate HCC cells growth, migration and colony formation *in vitro* and enhance HCC tumorigenicity, growth and lung metastasis *in vivo*. The stability of suppressor of cytokine signaling 2 (SOCS2) mRNA can be downregulated via YTHDF2. And SOCS2 mRNA is a downstream target of METTL3 and a tumor inhibitor in HCC. Particularly, as a clinical manifestation of HCC, the higher level of METTL3 might predict a poor prognosis. RBM15B, KIAA1429 and m6A level on mRNA also increase in HCC while METTL14 has no significant change. However, the disturbance of METTL14 obviously alters the proliferation, migration and colony formation of Huh-7 cell (Chen et al., 2017b). Interestingly, in another study, METTL14 and FTO decreased in HCC while METTL3, WTAP, KIAA1429, and ALKBH5 have no remarkable change. The downregulation of METTL14 acts as a poor prognostic indicator for survival without recurrence in HCC and has a close association with tumor metastasis. Specifically, METTL14 can promote pri-miR126 processing to mature miR126, a tumor suppressor in HCC metastasis, by mediating the recognition and binding of the microprocessor protein DGCR8 to pri-miRNA (Ma et al., 2017).

YTHDF2 is closely associated with the malignance of HCC. YTHDF2 regulates mRNA degradation by recognizing mRNA m6A sites, leading to the enhancement of proliferation of HCC cells. miR-145, which is down-regulated in HCC patients, can suppress the expression of YTHDF2 by directly targeting the 3′UTR of YTHDF2 mRNA(Yang et al., 2017). Thereby, miR-145 could be a candidate target for the treatment of liver cancer.

#### Cholangiocarcinoma (CCA)

Being diagnosed at advanced stage, CCA has a poor prognosis and limited curative options, and the overall survival rate is rather low. Invasion and migration cancer cells make CCA worse (Jo et al., 2013; Goldaracena et al., 2017; Kennedy et al., 2017).

As a nuclear protein, WTAP upregulates in CCA and has a positive correlation with TNM stage, lymph node metastasis and vascular invasion. WTAP siRNA inhibits CCA cells migration, invasion and tumorigenicity rather than proliferation. The results of cDNA microarray and real time PCR have revealed that WTAP could promote metastasis-related genes expression, such as MMP7, MMP28, and Muc1 (Jo et al., 2013). However, it is not clear whether WTAP function is related to m6A methyltransferase in CCA. Further investigations remain to be done.

### Breast cancer

In breast cancer, METTL3, HBXIP (mammalian hepatitis B X-interacting protein) and let-7g miRNA form a positive feedback loop (HBXIP/let-7g/METTL3/HBXIP) to promote cell proliferation (Figure 4). HBXIP enhances the expression of METTL3 by suppressing the tumor suppressor let-7g. The increase of METTL3, in turn, enhances the level of HBXIP by facilitating m6A modification in mRNA (Cai et al., 2018).

Hypoxia is a vital feature of the tumor microenvironment that drives cancer progression. With hypoxia induction, hypoxia inducible factor (HIF) dependent ALKBH5 strengthens pluripotency factor NANOG mRNA stabilization and accumulation by demethylation (Figure 4). Moreover, elevation of ALKBH5 increases the expression of NANOG in breast cancer stem cells (Zhang et al., 2016a). Furthermore, depletion of ALKBH5 or ZNF217, an m6A methyltransferase inhibitor, suppresses NANOG and Kruppel-like factor 4 (KLF4) expression by increasing m6A-methylated RNA (Zhang et al., 2016b). Of note, depletion of ALKBH5 inhibits tumor formation, decreases the breast cancer stem cells and suppresses metastasis from breast to lung in immunodeficient mice (Zhang et al., 2016a,b). The hypoxic induction of pluripotency factor, ALKBH5 and ZNF217 expression depends on HIF, meaning that HIF is vital for breast cancer therapy.

### Renal cell carcinoma (RCC)

METTL3 plays an inhibitory role in RCC and the higher level indicates a better prognosis. The expression levels of METTL3 mRNA and protein decline in RCC. Depletion of METTL3 promotes cell proliferation, growth, and colony formation through PI3K-Akt-mTOR pathway activation, and enhances cell migration and invasion by epithelial-mesenchymal transition (EMT) pathway. METTL3 knockdown also decreases cell cycle arrest in G1 phase and P21 expression, which acts as a suppressor in tumor (Li et al., 2017b). In a word, METTL3 exerts an essential role in the progression of RCC (Figure 4).

### Pancreatic cancer

High expression of YTHDF2 mRNA and protein in pancreatic cancer have a positive correlation with its progression (Figure 4). In pancreatic cancer cells, YTHDF2 serves as a suppressor in adhesion, invasion, migration and EMT through YAP signaling, but YTHDF2 acts as a promoter in proliferation via Akt/GSK3b/CyclinD1 pathway (Chen et al., 2017a). As a potential diagnostic and prognostic marker, whether YTHDF2 could be a new therapeutic target remains to be elucidated.

### Colon cancer

RNA helicase YTHDC2 acts as a promoter in colon cancer metastasis by enhancing the translation of hypoxia-inducible factor-1alpha (HIF-1α) gene, which could promote EMT via the transcription factor Twist1 (Figure 4). Further, the 5′UTR of both HIF-1α and Twist1 are the targets of YTHDC2 under hypoxia. YTHDC2 upregulates in colon cancer and has an obviously positive correlation with tumor stage, indicating YTHDC2 may be a diagnostic marker for colon cancer patients (Tanabe et al., 2016). YTHDC2 serves as a RNA helicase rather than the reader of m6A in this study (Tanabe et al., 2016), although other studies have certified that YTHDC2 is a N6-methyladenosine binding protein (Hsu et al., 2017). Whether the two kinds of YTHDC2 belong to the same substance and the exact mechanism still need to be explored.

### Cervical cancer

In cervical cancer, decreased m6A is closely related to tumor size, FIGO stage, differentiation, lymph node infiltration and tumor recurrence (Figure 4). The reduction of m6A plays a positive role in cell proliferation and tumor development (Wang et al., 2017b). In particular, FTO might upregulate both mRNA and protein expression of β-catenin, interacting with excision repair cross-complementation group 1 (ERCC1). Ultimately, this process leads to resistance of chemo-radiotherapy on cervical squamous cell carcinoma, the major type of cervical cancer. More importantly, although FTO expression is not associated with the 5-year overall survival, the patients with high FTO and β-catenin expression has a poor prognosis (Zhou et al., 2018). The findings of FTO function in response to the treatment of cervical cancer expands our understanding of m6A role in cancer development.

## Conclusions and outlooks

RNA epigenetics has become a hot topic in recent years. Among more than 100 kinds of different chemical modifications, m6A is the most abundant modification with the feature of dynamic and reversibility. It is installed by “writers” and removed by “erasers.” “Readers” are the m6A recognition proteins. m6A is extremly important for mRNA metabolism at different stage, from processing in the nucleus to translation and decay in the cytoplasm. Besides, m6A regulates circadian rhythm, cell cycle, cell differentiation, reprogramming, state transitions and stress responses (Zhao et al., 2017). Except for m6A, other chemical modifications also are irreplaceable, such as m1A, m5C, 2′-O-methylation (2'OMe), pseudouridine (ψ) (Esteller and Pandolfi, 2017; Zhao et al., 2017). For example, m5C plays a significant role in translation efficency, mRNA structure, genetic recoding of coding gene. It also regulates vault ncRNA process into small RNA and tRNA cleavage (Esteller and Pandolfi, 2017).

Diverse cancers are influenced by the structures and functions of m6A modification. In this review, we summarized the mechanisms and functions of m6A-modified RNA in 10 cancers, including AML, GBM, lung cancer, HCC, CCA, breast cancer, RCC, pancreatic cancer, colon tumor and cervical cancer (Table 1, Figures 2–4). Compared to m6A “writers” and “erasers,” only few articles studied the relationship between m6A “readers” and cancers. YTHDF2 in GBM, HCC, intestinal-type gastric adenocarcinoma and lung adenocarcinoma is upregulated. Genetic alterations of m6A readers indicates a poor survival in AML (Kwok et al., 2017). However, the underlying mechanism of m6A “readers” in cancers still remains a mystery. Meanwhile, other modifications also play a vital role in cancers. For instance, ALKBH3, a m1A RNA demethylase, can protect the genome against alkylation damage. TET1, a RNA m5C demethylase, acts as a tumor suppressor in leukemia (Esteller and Pandolfi, 2017). Whether there is joint effect or subtractive effect between m6A and other modifications remains to be further studied.

**Table 1:** Clinical significance of the regulators of m6A in different cancers.

**Cancer**	**m6A Regulators**	**Change**	**Sample Source**	**Clinical Significance**	**References**
AML	METTL3, METTL14, WTAP, FTO	Up	Patients, nude mice, AML cells	Therapy	^a^
GBM	METTL3 METTL14/METTL3 WTAP ALKBH5	Up Down Up Up	Tissue, nude mice, GSCs NSG mice, GSCs U87MG/ GBM05 cell, nude mice, tissue Tissue, Foxn1^nu/nu^ nude mice, GSCs	Therapy Therapy Prognosis Prognosis	^b^
Lung Cancer	METTL3	Up Down	A549/H1299/H1792/HEK cell Tissue, A549/NCI-H460 cell	NA Therapy	Lin et al., 2016; Du et al., 2017
HCC	METTL3 METTL14 YTHDF2	Up Down Up	Tissue, HepG2/Huh-7 cell, BABL/cAnN-nude mice Tissue, HepG2 cell, BALB/c nude mice Tissue, HepG2 cell	Prognosis Prognosis Prognosis	Chen et al., 2017b; Ma et al., 2017; Yang et al., 2017
CCA	WTAP	Up	Tissue, HuCCT1/SUN196 cell, nude mice	NA	(Jo et al., 2013)
Breast Cancer	METTL3 ALKBH5	Up Up	Tissue, MCF-7 cell MCF-7/MDA-MB-435/T4TD cell, NSG mice	NA Therapy	^c^
RCC	METTL3	Down	Tissue, CAKI-1/CAKI-2/ACHN cell, BALB/c nude mice	Diagnosis, prognosis	Li et al., 2017b
Pancreatic cancer	YTHDF2	Up	Tissue, SW1990/PaTu8988/BxPC3 cell	Diagnosis, prognosis	Chen et al., 2017a
Colon Tumor	YTHDC2	Up	HT29/HCT116/COS cell, BALB/c ^nu/nu^ mice	Diagnosis, therapy	Tanabe et al., 2016
Cervical Cancer	FTO	Up	Tissue, SiHa/c-33a cell, nude mice	Therapy, prognosis	Zhou et al., 2018

NA, Not Available.

a Vu et al. (2017), Barbieri et al. (2017), Weng et al. (2017), Bansal et al. (2014), Li et al. (2017c), and Su et al. (2018).

b Xi et al. (2016), Cui et al. (2017), Visvanathan et al. (2018), Jin et al. (2012), and Zhang et al. (2017).

c *Cai et al. (2018), Zhang et al. (2016a), and Zhang et al. (2016b)*.

Among the regulators of m6A, METTL3, the crucial methyltransferase of m6A, plays a promotor in most of cancers. In colorectal, prostate and bile duct cancers, METTL3 has been reported to be significantly upregulated based on bioinformatic analysis (Chen et al., 2017b). In addition to methyltransferase activity, METTL3 also influences cancers indenpendently of its catalytic subunit. This typical function is to enhance the translation of oncogenes (Lin et al., 2016).

Insterestingly, the methyltransferases and demethylases have distinct impacts on various cancer cells. In AML cell, both of them promote cell proliferation and suppress cell differenation (Bansal et al., 2014; Li et al., 2017c; Vu et al., 2017; Weng et al., 2017). In GSCs, METTL3 and METTL14 suppress differenation while WTAP and ALKBH5 promote cell proliferation, self-renewal and tumorigenicity (Jin et al., 2012; Cui et al., 2017; Zhang et al., 2017; Visvanathan et al., 2018). The roles of METTL3 in lung cancer are inconsistent across different studies (Lin et al., 2016; Du et al., 2017). Elevated METTL3 palys an oncogenic role in lung cancer (Lin et al., 2016), while METTL3 is downregulated in NSCLC (Du et al., 2017). It is worth noting that alteration of m6A levels in HCC development is discordant across different studies, METTL3 and METTL14 show a completely contrary effects on migration of HCC cells (Chen et al., 2017b; Ma et al., 2017). These results may suggest that some functions of METTL3 are independent of m6A modification and the underlying mechanism remains to be explored.

Despite the roles of m6A in cancers have dramatically advanced in recent years, a large number of challenges still exist. First, the mechanisms of m6A regulators in some cancers are largely unkown. Second, if m6A level and its regulators could be potential biomarkers for diagnosis and prognosis of some caners, and the specificity and sensitivity of these biomarkers need to be explored. Third, a number of studies have suggested that m6A regulators and related pathways could be used as therapeutic targets, but lack of the specific applications in clinical practice with a large sample size, and the corresponding side effects are largely unkown. All of these issues should be clearly addressed.

## Author contributions

Z-XL and L-ML are the co-first author who writer the article. S-ML and H-LS are corresponding author.

### Conflict of interest statement

The authors declare that the research was conducted in the absence of any commercial or financial relationships that could be construed as a potential conflict of interest.
